# Supplementary material for: Mouse and human antibodies bind HLA-E-leader peptide complexes and enhance NK cell cytotoxicity
Source: Commun Biol. 2022 Mar 28;5:271. doi: 10.1038/s42003-022-03183-5 (PMC8960791; doi:10.1038/s42003-022-03183-5)
Supplement: Supplementary file 2 — Supplementary Information [file 42003_2022_3183_MOESM2_ESM.pdf]

Supplementary Materials for

Mouse and Human Antibodies Bind HLA-E-Leader Peptide Complexes and Enhance NK Cell Cytotoxicity

Dapeng Li[1,2,#], Simon Brackenridge[3,#], Lucy C. Walters[3,#], Olivia Swanson[1], Karl Harlos[4], Daniel Rozbesky[4,5], Derek W. Cain[1,2], Kevin Wiehe[1,2], Richard M. Scearce[1], Maggie Barr[1], Zekun Mu[1], Robert Parks[1], Max Quastel[3], Robert J. Edwards[1,2], Yunfei Wang[1,2], Wes Rountree[1,2], Kevin O. Saunders[1,6,7], Guido Ferrari[7], Persephone Borrow[3], E. Yvonne Jones[4], S. Munir Alam[1,2], Mihai L. Azoitei[1,2,*], Geraldine M. Gillespie[3,*], Andrew J. McMichael[3,*], Barton F. Haynes[1,6,*]

[1]Duke Human Vaccine Institute, Duke University School of Medicine, Durham, NC 27710, USA

[2]Department of Medicine, Duke University School of Medicine, Durham, NC 27710, USA

[3]Nuffield Department of Clinical Medicine, University of Oxford, Oxford, OX3 7FZ, UK

[4]Division of Structural Biology, Wellcome Centre for Human Genetics, University of Oxford, Oxford, OX3 7BN, UK

[5]Department of Cell Biology, Charles University, Prague, 12800, Czech Republic

[6]Department of Immunology, Duke University School of Medicine, Durham, NC 27710, USA

[7]Department of Surgery, Duke University School of Medicine, Durham, NC 27710, USA

[#]Authors contributed equally

*Address correspondence to mihai.azoitei@duke.edu, geraldine.gillespie@ndm.ox.ac.uk, andrew.mcmichael@ndm.ox.ac.uk and barton.haynes@duke.edu

SUPPLEMENTAL FIGURES

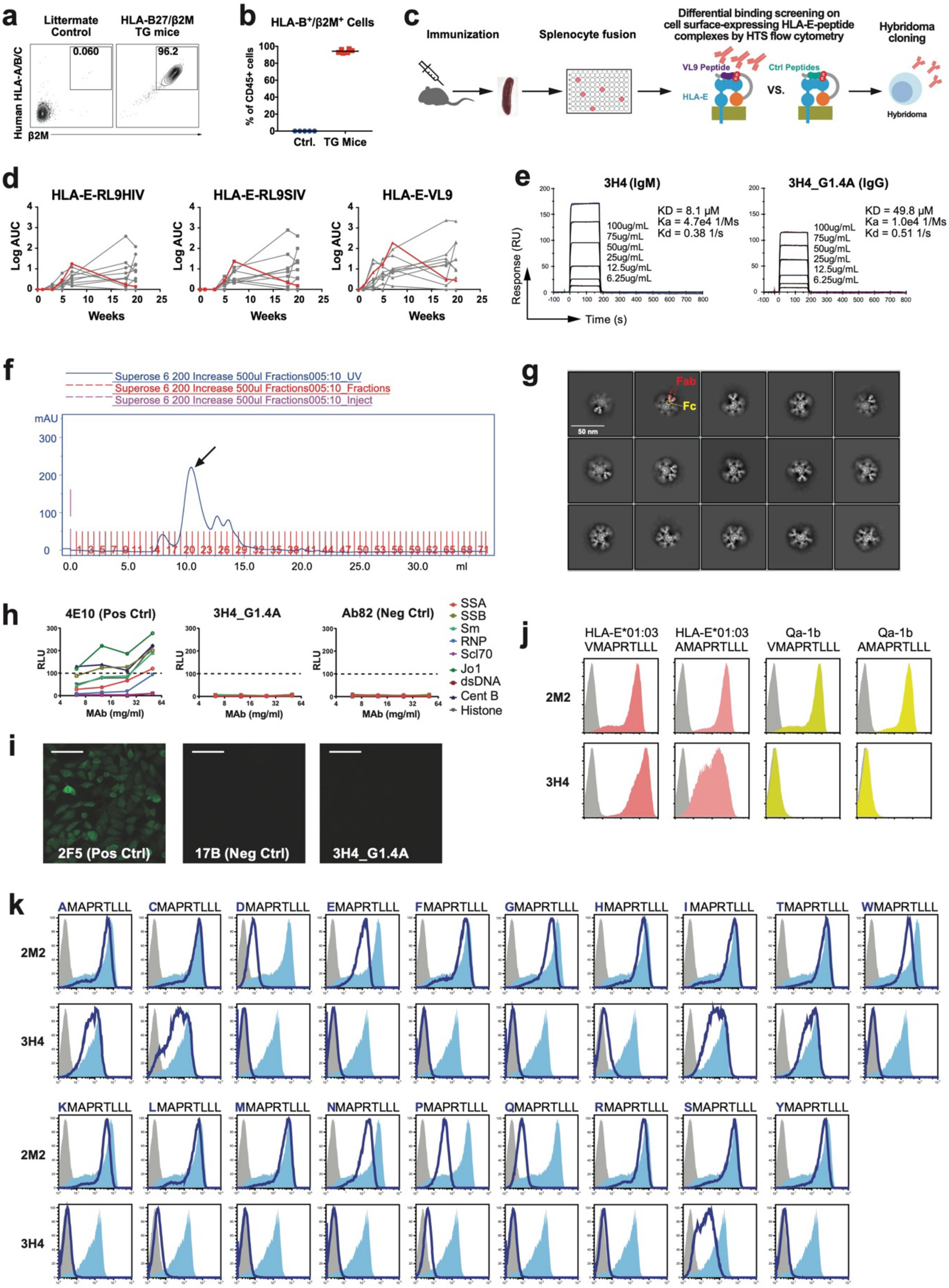

Supplementary Figure 1. Isolation and characterization of monoclonal antibody 3H4.

a-b, Expression of human HLA-B27 and β2M in peripheral blood lymphocytes (PBLs) of the transgenic (TG) mice. HLA-B27/β2M TG mice were used to minimize the induction of antibodies to HLA class I and β2M. Mouse PBLs from TG mice and littermate control were isolated and stained with anti-mouse CD45, anti-human HLA class I (A/B/C) and anti-human β2m antibodies. Representative data (a) and the percentages of human HLA-B27^{+}β2M^{+} cells in CD45^{+} PBLs from TG mice (n=6) and control mice (n=6) (b) were shown.

c, Schematic diagram of the immunization, splenocyte fusion and hybridoma screening strategy. HLA-B27/β2M TG mice (n=10) were immunized with cell surface-expressing HLA-E-RL9 peptide (a peptide derived from HIV-1; denoted RL9HIV hereafter) single-chain trimer (SCT)-transfected 293T cells (indicated by red arrows). After immunizations, spleen cells were harvested from the selected mouse and the fusion was performed using NS0 cells to generate hybridoma cells. Supernatants from the hybridoma cell candidates were screened for differential binding by surface staining on HLA-E-VL9, HLA-E-RL9HIV or HLA-E-RL9SIV transfected 293T cells. Hybridomas producing antibodies specific for HLA-E-VL9 but not others were selected for cloning and downstream analysis. Monoclonal cells were cloned for at least five rounds.

d, Serum antibody binding ELISA. Serum antibodies to HLA-E-VL9, HLA-E-RL9SIV, HLA-E-RL9HIV complexes were quantified by ELISA and shown as log AUC (area under curve). Antigens used for immunizations and ELISA assays are all cysteine (C)-trap stabilized. Each curve represents one animal, and the curve for animal that we used for splenocyte fusion are shown in red.

e, Affinity of 3H4 binding to soluble HLA-E-VL9 complex. 3H4 as a mouse IgM or as a recombinant human IgG1 were immobilized on CM5 sensor chips and soluble HLA-E-VL9 complex protein at the indicated concentrations was flowed over antibody immobilized sensor chips. Binding data are shown as black lines, and the best fits of a 1:1 binding model are shown as colored lines. Rate constants (k_a, k_d) and dissociation constant K_D were measured following curve fitting analysis.

f, Purification of 3H4 by FPLC using Superose 6 size exclusion column. The arrowed peak was collected and analyzed by negative staining.

g, Representative class average images of 3H4 negative stain electron microscopy (NSEM).

h, Binding of 3H4 expressed in a human backbone G1.4A to a panel of autoantigens by AtheNA assays. HIV-1 gp41 antibody 4E10 was set as a positive control, and a Flu antibody Ab82 was used as a negative control. The dotted lines indicate the cutoff values ≥100 luminance units used to denote positivity.

i, Binding of 3H4 in human backbone G1.4A to HEp-2 epithelial cells in indirect immunofluorescence staining assays. HIV-1 gp41 antibody 2F5 was set as a positive control, and HIV-1 gp120 antibody 17B was used as a negative control. Antibody staining concentration was 50 μg/ml, and data were collected at 40x objective for 8 seconds. Scale bar: 100 μm. Data are representative from one of two independent experiments.

j, 3H4 does not cross-react with mouse Qa-1b-peptide complex. 293T cells were transfected with HLA-E-VL9 (VMAPRTLLL), HLA-E-AL9 (AMAPRTLLL), mouse Qa-1b-VL9, or mouse Qa-1b-AL9. Transfected cells were stained with 3H4 antibody or an anti-β2M control antibody 2M2 followed by AF647 conjugated anti-mouse

IgG(H+L) secondary antibody. Data are representative from one of three independent experiments.

k, 3H4 recognizes peptides with variants in P1. 293T cells were transfected with HLA-E SCTs with VL9 peptides with single amino acid mutations at P1, then stained with 3H4 antibody or an anti-β2M control antibody 2M2 followed by AF647 conjugated anti-mouse IgG(H+L) secondary antibody (dark blue). Cells were gated for EGFP positive subsets. Isotype control stained cells were used as a negative control (grey filled histograms), and the wildtype VL9 peptide was a positive control (pale blue filled histograms). Data are representative from one of three independent experiments.

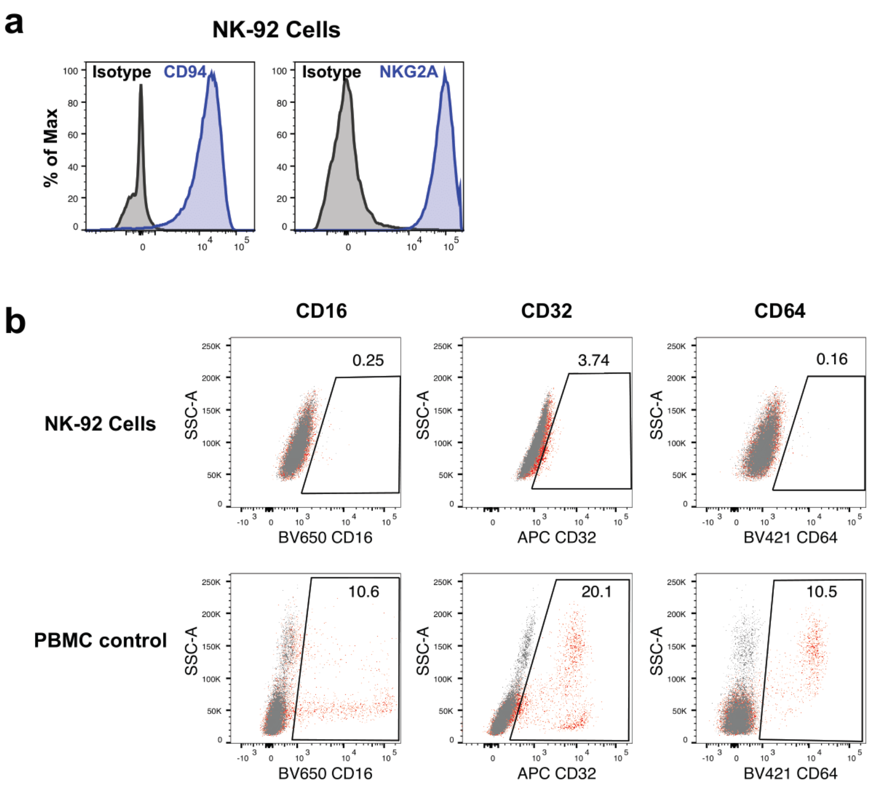

Supplementary Figure 2. Phenotypic analysis of NK-92 cells.

a, NKG2A and CD94 expression in NK-92 cell line detected by flow cytometry. NK-92 cells were stained with PE-CD94 antibody or FITC-NKG2A antibody and analyzed in flow cytometer. A PE-isotype and FITC-isotype antibodies were used as negative controls.

b, Fc receptors CD16, CD32 and CD64 expression in NK-92 cell line detected by flow cytometry. NK-92 cells were stained with BV650-CD16 antibody, APC-CD32 antibody, or BV421-CD64 antibody and analyzed in flow cytometer. Peripheral blood mononuclear cells (PBMCs) were used as positive controls. Dot plots overlay of antibody stained cells (red) and unstained control cells (grey) were shown. NK-92 cells were negative for CD16, CD32 or CD64, while a subset of PBMC cells were positive for each antibody. Data from a single antibody phenotype experiment.

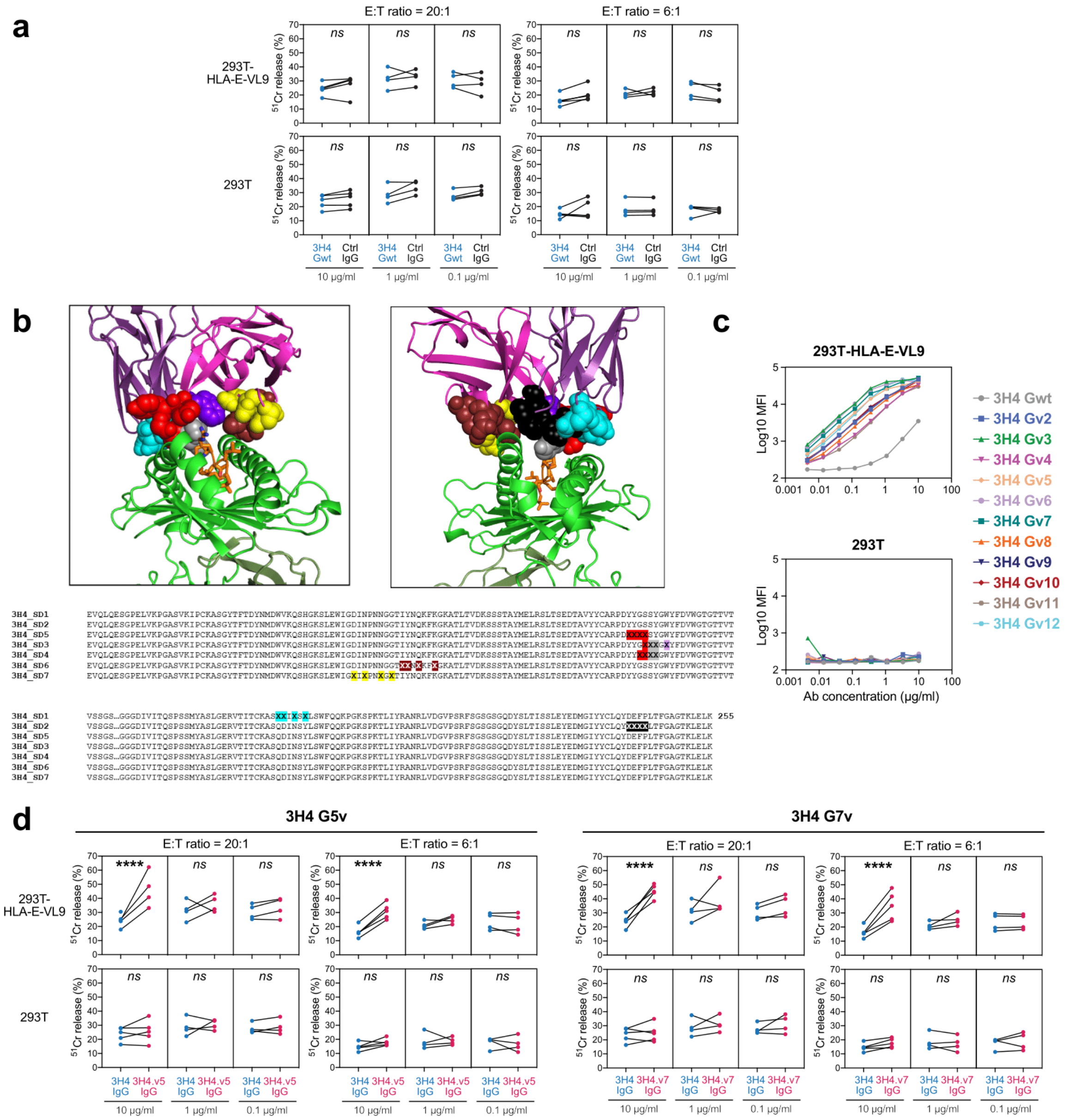

Supplementary Figure 3. Affinity optimization of 3H4 IgG.

a, NK cell cytotoxicity against wild-type 3H4 IgG-treated target cells measured by ^{51}Cr release assay. Unoptimized, wild-type 3H4 on a human IgG1 backbone (3H4 Gwt) was incubated with HLA-E-VL9 transfected 293T cells and untransfected 293T cells at final concentration of 10 µg/ml, 1 µg/ml, or 0.1 µg/ml, and NK92 cells were added into the mixture as effector cells at effector: target (E:T) ratios of 20:1 and 6:1. Human IgG1 CH65 was used as an isotype controls. Dots represent the

mean values of triplicate wells in four or five independent experiments. Asterisks show the statistical significance between indicated groups: ns, not significant.

b, 3H4 residues optimized for affinity improvements by library screening. Seven different libraries were designed that simultaneously sampled group of 4 amino acids in the CDR loops of 3H4 that interact with HLA-E-VL-9 by structural analysis. *Top*: Structural mapping of the amino acids (*spheres*) sampled together in the different libraries. Residues shown in the same color were randomized together. HLA-E: *green*; VL-9: *orange*; *Bottom*: Amino acid sequence of the seven 3H4 libraries, with randomized residues marked with '*X'* and colored as in the structural panels above.

c, Binding of wild-type 3H4 and variants on transfected 293T cells. Wild-type 3H4 and variants were titrated on HLA-E-VL9-transfected or untranfected 293T cells. Mean fluorescent intensity (MFI) from one of three independent experiments were shown.

d, Enhanced NK-92 cell cytotoxicity by optimized IgG 3H4 Gv5 and 3H4 Gv7 on HLA-E-VL9 transfected 293T cells and untransfected 293T cells, in compare with IgG 3H4 Gwt. Dots represent the mean values of triplicate wells in four or five independent ^{51}Cr release assays. Statistical analysis was performed using mixed effects models. Asterisks show the statistical significance between indicated groups: ns, not significant, *P<0.05, **P<0.01, ***P<0.001, ****P<0.0001.

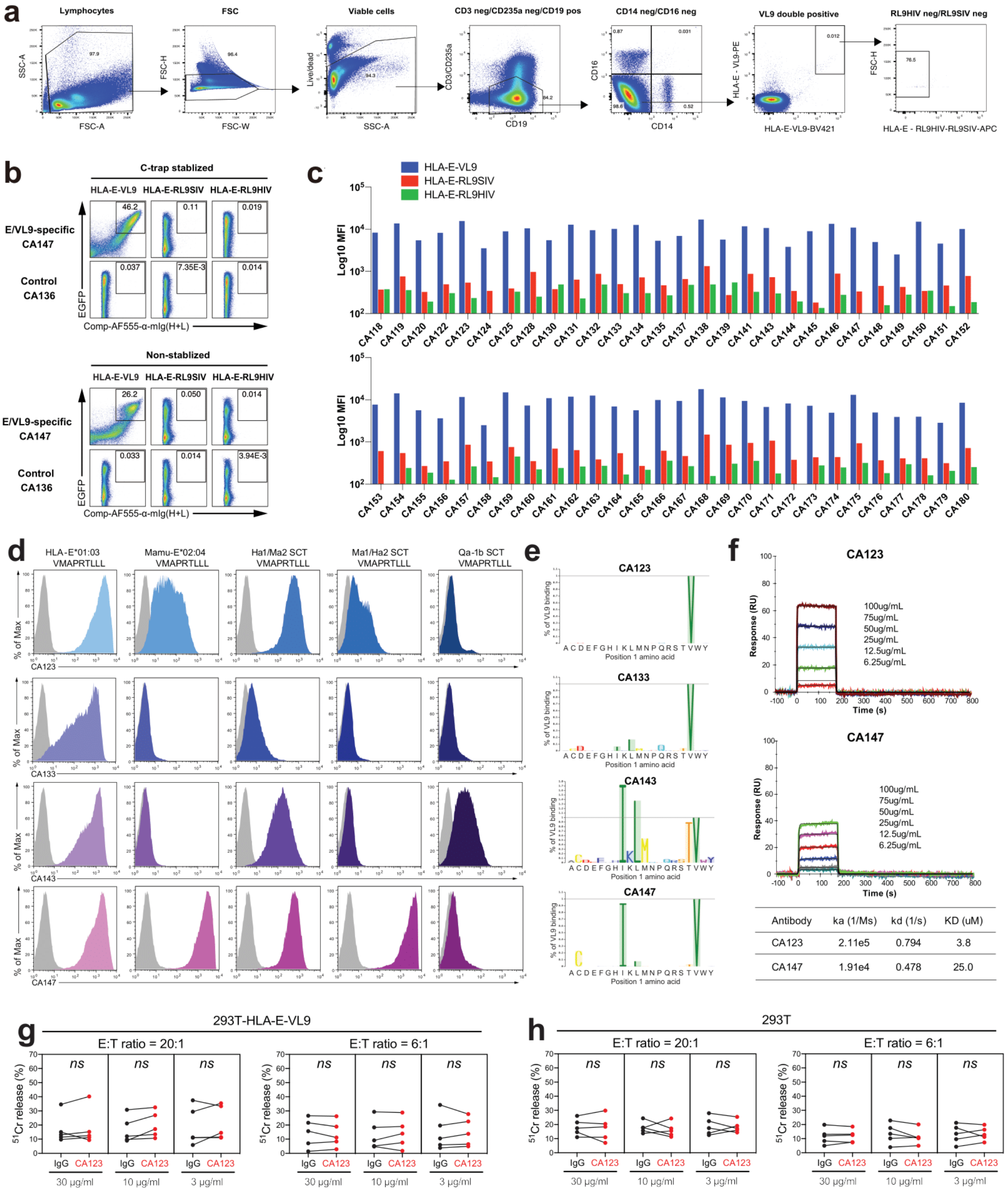

Supplementary Figure 4. Isolation and characterization of human HLA-E-VL9-specific antibodies.

a-b, HLA-E-VL9-specific B cell phenotyping from mice. Splenic cells isolated from HLA-B27/β2M TG mice (a) or C57BL/6 mice (b) were stained and analysed

flow cytometry for B cells (B220+CD19+) that are HLA-E-VL9 double positive, HLA-E-RL9HIV negative and HLA-E-RL9SIV negative.

c, Gating strategy of the single cell sorting for HLA-E-VL9-specific B cells from a Cytomegalovirus (CMV)-negative, male human. Human B cells were first enriched from PBMCs by pan-B cell negative selection magnetic beads. The enriched cells were stained and gated on viable/CD14^{neg}/CD16^{neg}/CD3^{neg}/CD235a^{neg}/CD19^{pos}/HLA-E-VL9^{pos}/HLA-E-RL9HIV^{neg}/ HLA-E-RL9SIV^{neg} subset as shown. Cells were single-cell sorted into 96-well plates for the downstream PCR cloning. Representative data from one of the four donors were shown.

d-e, Flow cytometry titration of purified HLA-E-VL9-specific mAbs isolated from a CMV-negative, male human. Antibodies recovered from sorted B cells were constructed in human IgG1 backbones and used for staining titration on both C-trap-stabilized and unstabilized HLA-E-VL9, HLA-E-RL9SIV, HLA-E-RL9HIV transfected 293T cells. EGFP expression indicates transfection efficiency. Transfected cells were stained with testing antibodies at the concentration of 2 μg/ml, followed by secondary antibody AF555-anti-human IgG staining. (d) Staining data of a representative antibody CA147 and a negative control antibody CA136. (e) Summary of the MFI of antibody binding data shown as bar chart. Data are representative from one of two independent experiments.

f, Cross-reactivities of human HLA-E-VL9 antibodies with rhesus Mamu-E-VL9 and mouse Qa-1b-VL9 complex. 293T cells were transfected with HLA-E-VL9, Mamu-E-VL9, two HLA-E/Mamu-E hybrids [HLA-E α1/Mamu-E α2 (Hα1/Mα2) and Mamu-E α1/HLA-E α2 (Mα1/Hα2)], and Qa-1b-VL9. Transfected cells were stained with human antibodies CA123, CA133, CA143, and CA147, followed by AF647

conjugated anti-mouse IgG(H+L) secondary antibody. Data are representative from one of three independent experiments.

g, Mapping of representative HLA-E-VL9-specific mAbs CA123, CA133, CA143 and CA147 on 293T cells transfected with HLA-E-VL9 peptide variants. 293T cells were transfected with HLA-E SCTs with VL9 peptides with single amino acid mutations at P1, then stained with human antibodies CA123, CA133, CA143, and CA147, followed by AF647 conjugated anti-mouse IgG(H+L) secondary antibody (dark blue). Cells were gated for EGFP positive subsets. MFI of the indicated antibody staining on wildtype VL9 peptide was set as 100%, and the percentages equals to (MFI of binding on each P1 variant) / (MFI of binding on wildtype VL9) x 100%.

h, Affinity measurements of human HLA-E-VL9 antibodies binding to soluble HLA-E-VL9 complex. Human antibodies CA123 or CA147 on human IgG1 backbone was immobilized on CM5 sensor chips and soluble HLA-E-VL9 complex protein at the indicated concentrations was flowed over the antibody immobilized sensor chips. Rate constants (k_a, k_d) and dissociation constant K_D were measured following curve fitting analysis.

i-j, NK cell cytotoxicity against CA123 IgG-treated target cells as assessed by ^{51}Cr release assay. Human antibody CA123 was incubated with HLA-E-VL9 transfected 293T cells (i) and untransfected 293T cells (j) at final concentration of 30 μg/ml, 10 μg/ml or 3 μg/ml, and NK92 cells were added into the mixture as effector cells at effector: target (E:T) ratio of 20:1 and 6:1. Human antibody A32 were used as the isotype control. Dots represent the mean values of triplicate wells in five independent experiments. Statistical analysis was performed using mixed effects models.

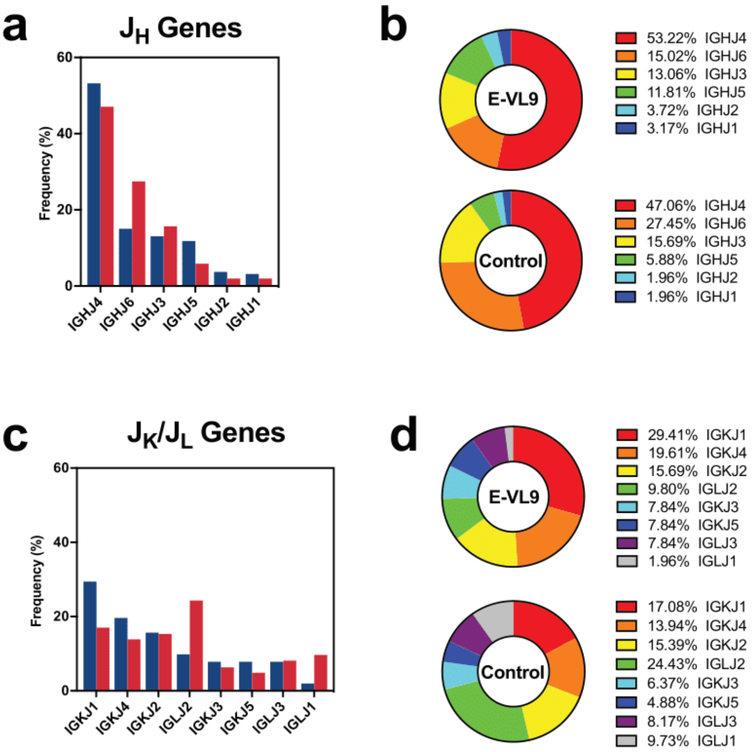

Supplementary Figure 5. J chain sequence analysis of HLA-E-VL9-specific antibodies (n=51). Reference VH-VL repertoires (n=198,148) from three healthy humans from a previous study (DeKosky et al., 2015) was used as a control.

a-b, Heavy chain (J_H) gene usage shown as bar chart (a) and pie chart (b).

c-d, Kappa chain (J_k) and lambda chain (J_L) gene usage shown as bar chart (c) and pie chart (d).
