# Supplementary material for: Mouse and human antibodies bind HLA-E-leader peptide complexes and enhance NK cell cytotoxicity
Source: Commun Biol. 2022 Mar 28;5:271. doi: 10.1038/s42003-022-03183-5 (PMC8960791; doi:10.1038/s42003-022-03183-5)
Supplement: Supplementary file 3 — Description of Additional Supplementary Files [file 42003_2022_3183_MOESM3_ESM.pdf]

Description of Additional Supplementary Files

File name: Supplementary Data 1

Description: Gene usage and mutation rate of four HLA-E-VL9-specific mAbs isolated from immunized transgenic mice.

File name: Supplementary Data 2

Description: Information of human subjects used in this study.

File name: Supplementary Data 3

Description: HLA-E-VL9-specific antibodies isolated from human. Index sorting MFI, immunogenetics information, and transfected 293T cell staining MFI of the 60 HLA-E-VL9-specific antibodies isolated from single B cell sorting were shown. The original isotypes of all the antibodies were IgMs. Antibodies from the same clonal family were highlighted in yellow.

File name: Supplementary Data 4

Description: The source data for the graphs and charts presented in the main figures.
