# Supplementary material for: Mouse and human antibodies bind HLA-E-leader peptide complexes and enhance NK cell cytotoxicity
Source: Commun Biol. 2022 Mar 28;5:271. doi: 10.1038/s42003-022-03183-5 (PMC8960791; doi:10.1038/s42003-022-03183-5)
Supplement: Supplementary file 8 — Reporting Summary [file 42003_2022_3183_MOESM8_ESM.pdf]

nature portfolio

Corresponding author(s): Dapeng Li

Last updated by author(s): Dec 7, 2021

Reporting Summary

Nature Portfolio wishes to improve the reproducibility of the work that we publish. This form provides structure for consistency and transparency in reporting. For further information on Nature Portfolio policies, see our Editorial Policies and the Editorial Policy Checklist.

Statistics

For all statistical analyses, confirm that the following items are present in the figure legend, table legend, main text, or Methods section.

n/a	Confirmed	
☐	☒	The exact sample size (*n*) for each experimental group/condition, given as a discrete number and unit of measurement
☐	☒	A statement on whether measurements were taken from distinct samples or whether the same sample was measured repeatedly
☐	☒	The statistical test(s) used AND whether they are one- or two-sided<br>*Only common tests should be described solely by name; describe more complex techniques in the Methods section.*
☒	☐	A description of all covariates tested
☐	☒	A description of any assumptions or corrections, such as tests of normality and adjustment for multiple comparisons
☐	☒	A full description of the statistical parameters including central tendency (e.g. means) or other basic estimates (e.g. regression coefficient) AND variation (e.g. standard deviation) or associated estimates of uncertainty (e.g. confidence intervals)
☐	☒	For null hypothesis testing, the test statistic (e.g. *F*, *t*, *r*) with confidence intervals, effect sizes, degrees of freedom and *P* value noted<br>*Give P values as exact values whenever suitable.*
☒	☐	For Bayesian analysis, information on the choice of priors and Markov chain Monte Carlo settings
☒	☐	For hierarchical and complex designs, identification of the appropriate level for tests and full reporting of outcomes
☒	☐	Estimates of effect sizes (e.g. Cohen's *d*, Pearson's *r*), indicating how they were calculated

Our web collection on statistics for biologists contains articles on many of the points above.

Software and code

Policy information about availability of computer code

Data collection	Diva<br>Biacore S200 Evaluation software
Data analysis	FlowJo v9.9.4<br>GraphPad Prism v8.3.1<br>SAS v9.4<br>Cloanalyst Program<br>MolProbity<br>Coot<br>Phenix<br>PyMOL Molecular Graphics System, version 2.0 (Schrödinger, LLC)

For manuscripts utilizing custom algorithms or software that are central to the research but not yet described in published literature, software must be made available to editors and reviewers. We strongly encourage code deposition in a community repository (e.g. GitHub). See the Nature Portfolio guidelines for submitting code & software for further information.

Data

Policy information about availability of data

All manuscripts must include a data availability statement. This statement should provide the following information, where applicable:

- Accession codes, unique identifiers, or web links for publicly available datasets
- A description of any restrictions on data availability
- For clinical datasets or third party data, please ensure that the statement adheres to our policy

The 3H4-HLA-E-VL9 co-complex structure determined in this study has the PDB accession code, 7BH8. The full datasets for human B cell repertoire analysis in the main figures are available in Supplementary Table 4; all other datasets generated during and/or analysed during the current study are available from the corresponding author on reasonable request.

Field-specific reporting

Please select the one below that is the best fit for your research. If you are not sure, read the appropriate sections before making your selection.

- [x] Life sciences
- [ ] Behavioural & social sciences
- [ ] Ecological, evolutionary & environmental sciences

For a reference copy of the document with all sections, see nature.com/documents/nr-reporting-summary-flat.pdf

Life sciences study design

All studies must disclose on these points even when the disclosure is negative.

Sample size	No statistical methods were used to predetermine sample size.
Data exclusions	No data was excluded.
Replication	All attempts at replication were successful in 3-6 independent experiments, as indicated in the figure legends.
Randomization	Samples were randomly allocated to groups prior to treatments which prevented any bias in the interpretation of data.
Blinding	The investigators were blinded in most of the assays but not in antibody isolation experiments. Functional assays were performed by laboratories independent from the discovery laboratory. No other data was supplied until after the assay was complete. Statistics were not calculated until the study was complete, and were done so by statisticians independent from the discovery researchers.

Reporting for specific materials, systems and methods

We require information from authors about some types of materials, experimental systems and methods used in many studies. Here, indicate whether each material, system or method listed is relevant to your study. If you are not sure if a list item applies to your research, read the appropriate section before selecting a response.

Materials & experimental systems

n/a	Involved in the study	
[ ]	[x]	Antibodies
[ ]	[x]	Eukaryotic cell lines
[x]	[ ]	Palaeontology and archaeology
[ ]	[x]	Animals and other organisms
[ ]	[x]	Human research participants
[x]	[ ]	Clinical data
[x]	[ ]	Dual use research of concern

Methods

n/a	Involved in the study	
[x]	[ ]	ChIP-seq
[ ]	[x]	Flow cytometry
[x]	[ ]	MRI-based neuroimaging

Antibodies

Antibodies used

PE-Cy5 Mouse Anti-Human CD3, Clone# HIT3a BD Biosciences Cat#555341; RRID: AB_10698936
BV605 Mouse Anti-Human CD14, Clone# M5E2 Biolegend Cat#301834, RRID: AB_2563798
BV570 Mouse Anti-Human CD16, Clone# 3G8 Biolegend Cat# 302035, RRID: AB_2632790
APC-Cy7 Mouse Anti-Human CD19, Clone# SJ25C1 BD Biosciences Cat# 557791, RRID: AB_396873
FITC Mouse Anti-Human IgD, Clone# IA6-2 BD Biosciences Cat# 555778, RRID: AB_396113
PerCp-Cy5.5 Mouse Anti-Human IgM, Clone# G20-127 BD Biosciences Cat# 561285, RRID:AB_10611998
PE-CF594, Mouse Anti-Human CD10, Clone# HI10A BD Biosciences Cat# 562396, RRID: AB_11154416
PE-Cy5 Mouse Anti-Human CD235a, Clone# GA-R2 BD Biosciences Cat# 559944, RRID: AB_397387

PE-Cy7 Mouse Anti-Human CD27, Clone# O323 eBioscience Cat# 25-0279, RRID: AB_1724039
APC-AF700 Mouse Anti-Human CD38, Clone# LS198-4-2 Beckman Coulter Cat# B23489, RRID: NA
SARS-CoV/SARS-CoV-2 Spike Ab, Clone# D001 Sino Biological Cat #40150-D001
Anti-influenza virus hemagglutinin human IgG CH65 (Whittle et al., 2011)
Rabbit polyclonal SARS-CoV-2 nucleocapsid Ab GeneTex Cat #GTX135357, RRID:AB_2868464
Alexa Fluor 555 (AF555) conjugated goat anti-mouse IgG (H+L) (Thermo Fisher, Catalog# A32727)
Alexa Fluor 647 (AF647) conjugated goat anti-mouse IgG (H+L) (Thermo Fisher, Catalog# A32728)
AF555 conjugated goat anti-human IgG (H+L) (Thermo Fisher, Catalog# A-21433)
AF647 conjugated goat anti-human IgG (H+L) (Thermo Fisher, Catalog# A-21445)
HRP goat anti-human IgG SouthernBiotech Cat #2040-05, RRID:AB_2795644
HRP goat anti-rabbit IgG Abcam Cat #ab97080, RRID:AB_10679808
Biotin mouse anti-human IgG Fc, Clone# H2 Southern Biotech Cat# 9042-08, RRID:AB_2796608

Validation

All antibodies are commercially available and are validated by the manufacturer (which can found via specific Brand and catalog numbers provided above) and in previous publications.

Eukaryotic cell lines

Policy information about cell lines

Cell line source(s)

ThermoFisher and the Farzan Laboratory at Scripps

Authentication

Each cell line is provided with a certificate of analysis. Cell identity is verified by morphology or fluorescent markers expressed.

Mycoplasma contamination

All cell lines undergo mycoplasma testing every 60 days.

Commonly misidentified lines
(See ICLAC register)

None to report.

Animals and other organisms

Policy information about studies involving animals; ARRIVE guidelines recommended for reporting animal research

Laboratory animals

Transgenic mice carrying human β2-microglobulin (β2m) and HLA-B*27:05 genes were obtained from Jackson lab (B6.Cg-Tg (B2M,HLA-B*27:05)56-3Trg/DcrJ; stock# 003428). Hemizygous mice were used in this experiment, as this strain is homozygous lethal. For hemizygous mice genotyping, peripheral blood lymphocytes (PBLs) were isolated and stained using mouse CD45 antibody (Biolegend, Catalog# 103122), human HLA class I antibody (Biolegend, Catalog# 311406) and human β2m antibody (Biolegend, Catalog# 316312).

Wild animals

No wild animals included.

Field-collected samples

No field-collected samples included.

Ethics oversight

All animal experiments were conducted with approved protocols from the Duke University Institutional Animal Care and Use Committee.

Note that full information on the approval of the study protocol must also be provided in the manuscript.

Human research participants

Policy information about studies involving human research participants

Population characteristics

Samples from four male donors were used in this study. Table S5 shows the clinical characteristics of the individuals studied.

Recruitment

Human leukapheresis frozen vials were collected by the External Quality Assurance Program Oversight Laboratory (EQAPOL) (Sanchez et al., 2014a; Sanchez et al., 2014b).

Ethics oversight

All experiments that related to human subjects was carried out with the informed consent of trial participants and in compliance with Institutional Review Board protocols approved by Duke University Medical Center.

Note that full information on the approval of the study protocol must also be provided in the manuscript.

Flow Cytometry

Plots

Confirm that:

- [x] The axis labels state the marker and fluorochrome used (e.g. CD4-FITC).
- [x] The axis scales are clearly visible. Include numbers along axes only for bottom left plot of group (a 'group' is an analysis of identical markers).
- [x] All plots are contour plots with outliers or pseudocolor plots.
- [x] A numerical value for number of cells or percentage (with statistics) is provided.

Methodology

Sample preparation	HLA-E SCT constructs encoding HLA-E-VL9, HLA-E-RL9HIV, or HLA-E-RL9SIV were transfected into 293T cells using GeneJuice transfection reagent (Novagen, Catalog# 70967). For epitope mapping experiment, a panel of HLA-E-VL9 SCT constructs with single amino acid mutations were transfected into 293T cells using the same method. Cells were dissociated with 0.1% EDTA at 48 hours post-transfection and stained with a Fixable Near-IR Dead Cell Stain Kit (Thermo Fisher, Catalog# L34976). After washing, primary antibodies (supernatant from hybridoma cells, supernatant from transfected cells, or purified antibodies) were added and incubated with cells for 1 hour at 4°C, following by staining with 1:1000 diluted secondary antibodies for 30 mins at 4°C. For mouse primary antibodies, we used Alexa Fluor 555 (AF555) conjugated goat anti-mouse IgG (H+L) (Thermo Fisher, Catalog# A32727) or Alexa Fluor 647 (AF647) conjugated goat anti-mouse IgG (H+L) (Thermo Fisher, Catalog# A32728) as secondary antibodies; for human primary antibodies, we used AF555 conjugated goat anti-human IgG (H+L) (Thermo Fisher, Catalog# A-21433) or AF647 conjugated goat anti-human IgG (H+L) (Thermo Fisher, Catalog# A-21445) as secondary antibodies. Cells were then washed 3 times and resuspended in fixation buffer (1% formaldehyde in PBS, pH7.4).
Instrument	Data were acquired on a BD LSR II flow cytometer or BD FACSAria II flow cytometer.
Software	Data were analyzed using FlowJo version 10.
Cell population abundance	Abundance of the cell populations of interest was determined by the appropriate negative control and the purity of sorted population was assessed by the post sort analysis
Gating strategy	FSC-A/SSC-A and SSC-H/SSC-W gates were applied to remove debris, and non-single cell aggregates respectively. For 293T cell staining, we gated on viable cells. For human B cell soring, HLA-E-VL9-specific B cells were sorted, we gated on viable CD3neg/ CD14neg /CD16neg /CD235aneg/CD19pos / HLA-E-VL9double-pos/ HLA-E-RL9HIVneg/HLA-E-RL9SIVneg subset as single cells in 96-well plates. Gating strategy is provided in the Supplementary Information.

- [x] Tick this box to confirm that a figure exemplifying the gating strategy is provided in the Supplementary Information.
